# Efficacy and safety of acupuncture for patients with chronic urticaria: study protocol of a randomized, sham-controlled pilot trial

**DOI:** 10.1186/s13063-019-3433-1

**Published:** 2019-06-04

**Authors:** Yunzhou Shi, Hui Zheng, Siyuan Zhou, Qianhua Zheng, Leixiao Zhang, Xianjun Xiao, Wei Cao, Ying Liu, Ying Li

**Affiliations:** Department of Acupuncture and Moxibustion, Chengdu University of Traditional Chinese Medicine, No. 37 Shierqiao Road, Jinniu District, Chengdu, 610075 Sichuan China

**Keywords:** Acupuncture, Chronic urticaria, Randomized controlled trial, Study protocol

## Abstract

**Background:**

Chronic urticaria (CU) is a refractory skin disease with long duration and a high recurrence rate. Acupuncture has been widely used for the treatment of CU in clinical practice in China. However, until now, there has been no appropriately designed randomized controlled trial (RCT) to provide explicit evidence about the effectiveness of acupuncture for the treatment of CU worldwide. Therefore, we plan to conduct a pilot study to explore its effectiveness and safety and determine the feasibility of studying acupuncture in a future, full-scale, RCT of CU.

**Methods/design:**

This randomized, sham-controlled, participant-blinded and assessor-blinded pilot trial is underway in China. A total of 60 participants with CU will be randomly assigned to two groups in a 1:1 ratio: one treated with real acupuncture and the other with sham acupuncture, for 10 sessions over 2 weeks. The experimental group will receive acupuncture on a fixed prescription of acupoints, whereas the control group will receive sham acupuncture, namely minimal acupuncture on non-acupuncture points. The primary outcome will be the urticaria activity score (UAS). The secondary outcomes will include a visual analogue scale (VAS) score for itching intensity, the Dermatology Life Quality Index (DLQI), the Hamilton Depression Scale (HAMD), the Hamilton Anxiety Scale (HAMA), the humoral immunity index, serum total IgE, and adverse events. The UAS, VAS for itching and the DLQI will be conducted at baseline and at 1, 2, 3, and 4 weeks after randomization. The HAMD, HAMA, humoral immunity index, and serum total IgE will be assessed at baseline and at 2 weeks after randomization. Adverse events will be summarized at 1 week and 2 weeks after randomization.

**Discussion:**

The pilot study mainly aims to investigate trial feasibility, and confirm basic information about its effects and safety. Results of this trial will help clarify whether the acupuncture treatment is beneficial for symptom improvement in patients with CU. The finding of this study will provide preliminary evidence on the effectiveness and safety of acupuncture for CU.

**Trial registration:**

Acupuncture-Moxibustion Clinical Trial Registry, AMCTR-ICR-18000190. Registered on 19 June 2018.

**Electronic supplementary material:**

The online version of this article (10.1186/s13063-019-3433-1) contains supplementary material, which is available to authorized users.

## Background

Urticaria is a condition characterized by the development of wheals (hives), angioedema, or both [1]. Angioedema in patients with urticaria is characterized by a sudden, pronounced erythematous or skin-colored swelling of the lower dermis and subcutis or mucous membranes, sometimes pain rather than itch, and slower resolution than that of wheals. Chronic urticaria (CU) is defined on presentation almost daily of transient wheals lasting more than 6 weeks in duration [2, 3]. CU is divided into two types: chronic spontaneous urticaria (CSU) and chronic inducible urticaria (CIU) [1]. CSU refers to the spontaneous appearance of wheals, angioedema or both for > 6 weeks due to known or unknown causes. The signs and symptoms of CIU are triggered by external specific factors, such as a mechanical stimulus (friction, pressure, and vibration), thermal stimulus (cold, heat), aquagenic stimulus (water), and electromagnetic stimulus (solar radiation) [4]. CSU may occur at any age. Recent research showed a female-to-male predominance of 2:1, with a prevalence between 0.5% and 1% [5]. In a meta-analysis [6], the prevalence of CIU was estimated at 13.1–14.9% among patients with CU. CU can induce misery and embarrassment and severely impair the quality of life [7–10], Evidence suggests that patients with CSU can substantially lose productivity at work, school, or in daily activities [11–13]. There are high direct and indirect healthcare costs for treating CU due to the large socioeconomic implications of a 20–30% reduction in performance [14].

The current treatment guidelines [1, 15] and consensus statement [3, 16] recommend a stepwise approach for the complete control of CU symptoms. The European Academy of Allergology and Clinical Immunology, the Global Allergy and Asthma European Network, World Allergy Organization (EAACI/GA^2^LEN/EDF/WAO) guidelines [1] recommend the use of second-generation H1-antihistamines as the first line of treatment. If no response is seen at the regular dose, the dose will be increased up to a 4-fold standard or licensed dose. If there is still no improvement, the guidelines recommend the use of omalizumab and cyclosporine A (CsA) as the third-line treatment. However, all H1-antihistamine treatment options, including the use of higher-than-standard doses, do not have an approved label for the treatment of CU, and many patients experience an inadequate response to most of these drugs [17]. Furthermore, the guidelines do not provide guidance on the choice, dose and duration of alternative treatment options in patients who remain symptomatic despite the use of H1-antihistamines. In addition, although omalizumab and cyclosporine A (CsA) have proven effective [18–20], they are expensive and can impose a serious economic burden on patients. Widespread use will depend on legal and economic factors [21]. Therefore, more and more patients have turned to seek non-pharmacological treatments.

In recent years, acupuncture as an antipruritic measure is used widely in the treatment of skin diseases such as eczema [22], urticaria [23, 24], and neurodermatitis [25]. Some systematic reviews [26, 27] have investigated the effectiveness of acupuncture in the management of CU, but the authors concluded that the clinical results presented in the reviews are tentative and should be interpreted cautiously due to lack of the quality of the studies included. Besides, its scientific basis is still relatively limited owing to a lack of adequate statistical power, insufficient reports, or relevant methodological shortcomings in previous studies. Therefore, we have designed a pilot study with a 2-week observation period to explore its effectiveness and safety and determine the feasibility of studying acupuncture in a future, full-scale, randomized controlled trial (RCT) of CU.

## Method and design

### Study design

This study is a randomized, sham-controlled, patient-assessor blinded, parallel-design clinical trial. Participants will be assigned to the experimental group or the control group for 10 sessions over 2 weeks. The experimental group will be treated with acupuncture in a fixed prescription of acupoints, and the control group will be treated with sham acupuncture, namely minimal acupuncture on non-acupuncture points.

### Setting of the study

**Fig. 1:**
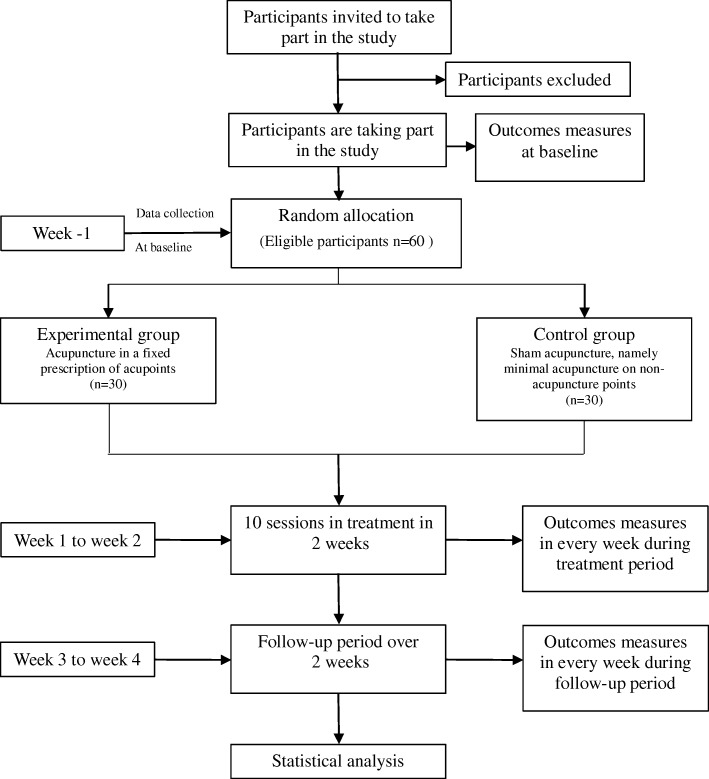
Flow diagram of the study design

This clinical trial will be conducted at the Affiliated Hospital of Chengdu University of Traditional Chinese Medicine. Eligible participants will be randomly assigned to the experimental group (*n* = 30) or the control group (*n* = 30) with a 1:1 ratio. The total observation period will be 5 weeks, including a 1-week baseline period, a 2-week treatment period and a 2-week follow-up period. They will receive 10 sessions of acupuncture or sham acupuncture treatment over 2 weeks (five sessions per week). During the baseline, treatment, and follow-up periods, participants will not be allowed to take any therapeutic medications (e.g., antihistamine, omalizumab, and CsA) for CU. but will be permitted to use antihistamines as necessary (e.g., loratadine, ebastine, and mizolastine) when the wheals and itching are unbearable. The number of wheals, degree of itching, impact on life, and medical history will be recorded in urticaria diaries every day. Assessments will be conducted at baseline and at 1, 2, 3, and 4 weeks after randomization. Figure 1 illustrates the study flowchart. The reporting of this trial is conducted according to the Standard Protocol Items: Recommendations for Intervention Trials (SPIRIT) guidelines [28] (Additional file 1), and the Standards for Reporting Interventions in Clinical Trials of Acupuncture (STRICTA) [29] have been used as frameworks of methodology for designing this protocol (Additional file 2).

### Participants

Prospective participants, who are diagnosed by two specialists (YH and PSH) in dermatology and fulfill all the inclusion criteria and meet none of the exclusion criteria, will be asked to talk face-to-face with research assistants to discuss the study and provide information on the eligibility criteria. Eligible patients who are interested in participating will be invited for a series of assessments of their condition and for safety assessments. Patients who give informed consent will be randomized into two groups receiving different treatments.

### Recruitment

Sixty patients with CU will be recruited through, but not limited to, reviewing and screening of outpatients in the Affiliated Hospital of Chengdu University of Traditional Chinese Medicine, university media releases, community advertisement including leaflets distributed via regular community health counseling, media campaigns, and network recruitment.

For subject recruitment, three research assistants will go to the department of dermatology in the Affiliated Hospital of Chengdu University of Traditional Chinese Medicine three times a week to identify and invite potentially eligible patients to participate in the study. The patients will be informed of the details of the study such as the objectives, scope, procedure, and potential benefits and risks. The patient will be given a written informed consent form. Patients or their carers will be informed of all the details and potential risks of the study, and the written informed consent form will be signed and witnessed by the research assistant. Oral informed consent may be acceptable if the participants cannot read.

### Inclusion criteria

Eligible participants should conform to EAACI/GA^2^LEN/EDF/WAO guidelines diagnostic criteria for CU and those who meet the following inclusion criteria will be included: (1) the sudden appearance of wheals, angioedema, or both; (2) the occurrence of spontaneous wheals, angioedema, or both for ≥ 6 weeks; (3) suffering from urticaria at least twice a week on average during the previous 6 weeks; (4) aged 18–70 years; (5) antihistamines were not used within 2 weeks before entering the study, and steroids hormone and immunosuppressive drugs were not used within 1 month; (6) agree to participate in this study and provide written informed consent.

### Exclusion criteria

Participants meeting any of the following criteria will be excluded: (1) urticaria is inducible by physical factors (e.g., cold urticaria, delayed pressure urticaria, solar urticaria, heat urticaria, vibratory angioedema); (2) suffering from neurological diseases, mental disorders, immunodeficiency, bleeding disorders; (3) having any serious disease of the heart, liver, kidney, or other organs; (4) those who are pregnant or lactating; (5) patients with severe cognitive impairment; (6) involved in other clinical studies at the same time.

### Withdrawal criteria

Participants meeting any of the following criteria will be withdrawn: (1) participant’s decision to drop out of the study at any time for any reason (2) occurrence of any unexpected serious side effects.

### Randomization, allocation concealment

A random number table will be generated with the computerized random number generator Package randomize R 3.5.1 (R Core Team (2013), R Foundation for Statistical Computing, Vienna, Austria). We will generate a random sequence list by using block and stratified design. Blocks will change from 2 to 4 and the sequence will be stratified by sex. The random number table will be prepared by an independent researcher (HZ) who will be responsible for assigning the recruited patients to the corresponding intervention code based on the list. He will not be involved in patient care, outcome assessment, data collection or data analysis. If the participants satisfy the inclusion criteria and sign the informed consent form, the acupuncture doctor who will conduct the acupuncture treatment will be informed of the group allocation.

### Blinding

All the participants, outcome assessors, and statisticians will be blinded to treatment allocation during the whole study. Due to the characteristics of the acupuncture clinical trial, it is not possible to blind the acupuncturists to treatment allocation in this trial. Therefore, they will not take part in the assessment procedure. Different treatment groups will receive treatment in different rooms to blind the participants to their treatment allocation. We will indirectly assess the success of blinding by testing credibility after all the treatment sessions [30], and participants will be asked to guess what kind of acupuncture treatment they have received.

### Interventions

In this trial, treatment strategies were developed by consensus with experienced acupuncture practitioners and dermatologists. There are two groups: active acupuncture group and sham acupuncture (superficial non-acupoint acupuncture) group. The location and manipulation of fixed acupoints and non-acupoints are shown in Table 1. Participants in both groups will receive 10 sessions of acupuncture over 2 weeks. Each session will be administered once a day for 5 days consecutively followed by a 2-day rest interval. All acupuncture treatment sessions will be performed by two acupuncturists who are registered Chinese Medicine Practitioners in China and have at least 3 years’ clinical experience in acupuncture practice. Acupuncturists will also receive training before this trial. Contents of the training classes cover the locating of acupoints, needle manipulation skills, and communication skills. The acupuncturists can be admitted to participate in the trial when they can pass the trial training examination.

**Table 1:** Locations and manipulations of the acupuncture points selected in this study

Group	Acupoints	Location	Manipulation
Acupoint	Baihui (GV20)	In the head, the front of the forehead is straight up to 5 cun	Subcutaneous insertion to a depth of 0.5–0.8 cun (12.5–20 mm) with manipulation for the *de-qi*
	Shenting (GV24)	In the head, the front of the forehead is straight up to 0.5 cun	Subcutaneous insertion to a depth of 0.5–0.8 cun (12.5–20 mm) with manipulation for the *de-qi*
	Quchi (LI11)	At the elbow, the midpoint of the lateral end of the elbow transverse line and the upper tibia	Puncture perpendicularly to a depth of 1–1.5 cun with manipulation for the *de-qi*
	Zhongwan (CV12)	In the upper abdomen, 4 cun above the umbilicus, the front midline	Puncture perpendicularly to a depth of 1–1.5 cun with manipulation for the *de-qi*
	Tianshu (ST25)	In the abdomen, parallel to the umbilicus, 2 cun in front of the front midline	Puncture perpendicularly to a depth of 1–1.5 cun with manipulation for the *de-qi*
	Xuehai (SP10)	In the anterior region of the femoral, 2 cun above the medial end of the patella and the protuberance of the vastus medialis	Puncture perpendicularly to a depth of 1–1.5 cun with manipulation for the *de-qi*
Non-acupoint	Zusanli (ST36)	Three inches below the patella between the anterior tibia and the extensor digit rum longus muscle	Puncture perpendicularly to a depth of 1–1.5 cun with manipulation for the *de-qi*
	Sanyinjiao (SP6)	On the medial side of the leg, 3 cun above the tip of the medial malleolus, posterior to the medial border of the tibia	Puncture perpendicularly to a depth of 1.0 cun with manipulation for the *de-qi.*
	Non-acupoint 1	Shangxing (GV23, in the head, the front of the forehead is straight up to 1 cun) opens 1 cun. Alternating left and right points	Subcutaneous insertion to a depth of 1–3 mm without manipulation for the *de-qi*
	Non-acupoint 2	Qianding (GV21, in the head, the front of the forehead is straight up to 3.5 cun) opens 1 cun. Alternating left and right points	Subcutaneous insertion to a depth of 1-3 mm without manipulation for *the de-qi*
	Non-acupoint 3	Ulnar side, halfway between the epicondyles medial to the humerus and ulnar side of the wrist	Subcutaneous insertion to a depth of 1–3 mm without manipulation for the *de-qi*
	Non-acupoint 4	Zhongwan (CV12, in the upper abdomen, 4 cun above the umbilicus, the front midline) opens 3 cm, alternating left and right points	Subcutaneous insertion to a depth of 1–3 mm without manipulation for the *de-qi*
Non-acupoint	Non-acupoint 5	Tianshu point (ST25 in the abdomen, parallel to the umbilicus, 2 cun in front of the front midline) opens 1 cun to the outside, the midpoint of the Tianshu and Daheng point (SP15, in the abdomen, parallel to the umbilicus, 4 cun in front of the front midline)	Subcutaneous insertion to a depth of 1–3 mm without manipulation for the *de-qi*
	Non-acupoint 6	On the thigh, the midpoint of the upper iliac spine and the upper outer corner of the sacral floor are opened inward to 2 cm	Subcutaneous insertion to a depth of 1–3 mm without manipulation for the *de-qi*
	Non-acupoint 7	Edge of the tibia 1–2 cm lateral to the Zusanli (ST36) horizontally	Subcutaneous insertion to a depth of 1–3 mm without manipulation for the *de-qi*
	Non-acupoint 8	3 cun above the outer ankle, between the stomach meridian and the gallbladder meridian	Subcutaneous insertion to a depth of 1–3 mm without manipulation for the *de-qi*

#### Acupuncture treatment group

Sterile, disposable, stainless steel needles of various lengths and diameters (Huatuo Medical Instruments Co. Ltd., Suzhou, China; 0.3 mm × 25 mm/0.3 mm × 40 mm) will be used in the intervention group. All acupoints will be punctured by filiform needles when patients are in a comfortable sitting position. The depth of these needles will be adjusted to the standard permissible layers of each acupoint. Then an even reinforcing-reducing technique, which means lifting, thrusting, twisting, and rotating the needles moderately, will be performed on the needles until achieving the *de-qi* sensation. The needles will be retained for 30 min in each session and manipulated every 10 min with intermittent stimulation to maintain the *de-qi* sensation. The manipulation of each acupoint will last for 10 s.

#### Sham-acupuncture treatment group

Participants assigned randomly to this group will be given superficial non-acupoint acupuncture 10 times over 2 weeks. The same type of acupuncture needle will be inserted perpendicular to the skin at a depth of 1.0–3.0 mm and the needles will be retained for 30 min without any manipulation to avoid the deqi sensation as much as possible. The number of acupuncture points, duration, and frequency of the sessions will be the same as those for the acupuncture treatment group, and the same practitioner will conduct the intervention.

### Outcome measurements

**Fig. 2:**
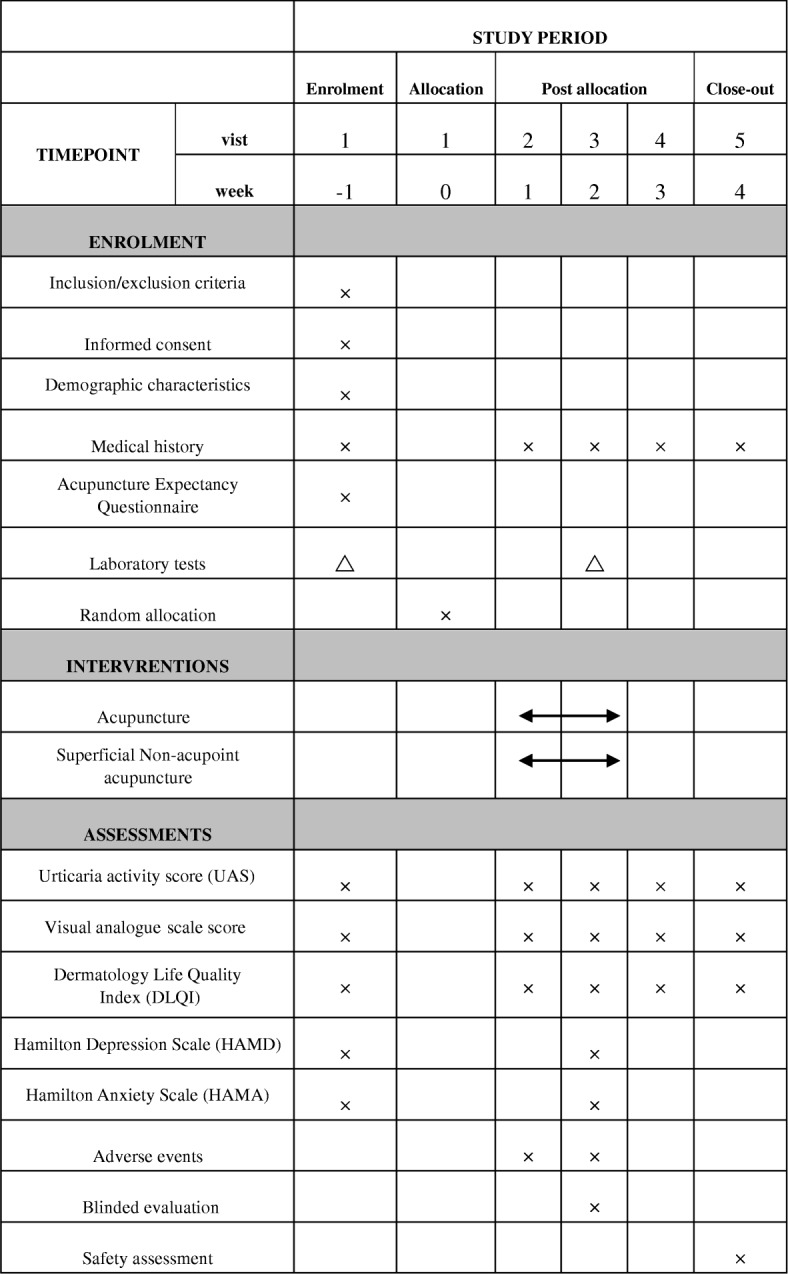
Timetable of treatment and outcome collection

The time schedule of enrolment, interventions, assessments, and visits of participants is shown in Fig. 2. The following outcomes will be assessed by independent assessors.

#### Primary outcome

The primary outcome measurement is the urticaria activity score (UAS) [1, 31]. The UAS, which is used to make daily assessments for 1 week, combines daily wheal numbers (0 = none, 1 = < 20 wheals/24 h, 2 = 20–50 wheals/24 h, and 3= > 50 wheals/24 h or large confluent areas of wheals) and pruritus intensity (0 = none, 1 = present but not annoying or troublesome, 2 = troublesome but does not interfere with normal daily activity or sleep, and 3 = severe pruritus, which is sufficiently troublesome to interfere with normal daily activity or sleep). It has a total score of 42 points for 1 week. The primary outcome measurement will be assessed at baseline and at 1, 2, 3, and 4 weeks after randomization.

#### Secondary outcome

##### Visual analogue scale (VAS) score of itching intensity per 1 week

The VAS is widely used for the assessment of variations in the severity of subjective symptoms. Participants mark their symptom severity on a 100-mm straight line whereby the extreme left end indicates “absence of symptoms” and the extreme right end indicates “unbearably severe symptoms” [32, 33]. The outcome measurement will be assessed at baseline and at 1, 2, 3, and 4 weeks after randomization.

##### Dermatology Life Quality Index (DLQI)

The DLQI is a compact questionnaire, which is applicable to individuals with any skin disease. It measures the influence of CU on the participant’s life over the previous 7 days. It consists of 10 questions with Likert-type responses and its score ranges from 0 to 30. Higher scores imply greater influence on health-related quality of life [34–36]. The outcome measurement will be assessed at baseline and at 1, 2, 3, and 4 weeks after randomization.

##### Hamilton Depression Scale (HAMD)

The HAMD comprises 17 items, which have been rated on a 3-point or 5-point scale. The HAMD total score ranges from 0 to 52. Clinicians can assess the intensity and frequency of depressive symptoms [37]. Higher scores imply greater depression (severe depression, score ≥ 24; presence of depression, score ≥ 17; possible depression, score ≥ 7; and no depression, score < 7). The outcome measurement will be assessed at baseline and at 2 weeks after randomization.

##### Hamilton Anxiety Scale (HAMA)

The HAMA is a 14-item clinical interview measure of somatic and psychic anxiety symptoms that have been rated on a 4-point scale [38]. The HAMA total score ranges from 0 to 56. Higher scores are positively correlated with higher levels of anxiety (severe anxiety, score ≥ 24; obvious anxiety, score ≥21, some degree of anxiety, score ≥ 14; possible anxiety, score ≥ 7; and no anxiety, score < 7). The outcome measurement will be assessed at baseline and at 2 weeks after randomization.

##### Humoral immunity index and measurement of serum total IgE

Although the clinical diagnosis of CU is straightforward, the etiology and pathogenesis of CU remain obscure. There is some evidence [39, 40] that the pathogenesis of CU is closely related to serum humoral immunity and serum total IgE. In this study, we will investigate the levels of serum immunoglobulins (IgG, IgA, and IgM), the complement components (C3 and C4), and serum total IgE before and after treatment in the two groups.

These laboratory tests will be measured using an enzyme-linked immunosorbent assay (ELISA). About 4 ml of whole blood will be collected from participants at two time points - at baseline and at 2 weeks after the completion of acupuncture treatment. Changes in various biomarker levels from baseline to 2 weeks will be examined in each of the treatment groups. Between-group differences will be analyzed to observe potential associations with the effect of acupuncture. These related tests will be conducted by the clinical laboratory of the Affiliated Hospital of Chengdu University of Traditional Chinese Medicine.

##### Adverse events

The participants will be requested to voluntarily report information about adverse events such as pallor, sweating or dizziness, fainting during acupuncture treatment, bleeding, local hematoma, unbearable prickling, and continuous severe pain more than 1 h after acupuncture. The acupuncturists will be requested to report adverse events related to the acupuncture such as sticking of the needle, broken needles, bent needles, and needles being retained after treatment. All adverse events will be fully recorded on the adverse event pages of the case report forms (CRFs). The researcher will confirm the occurrence of adverse events and record all details such as the date of occurrence, time, degree, measurement related to the treatment, and causal relationship with the treatment. Emergency medical assistance will be sought if any serious adverse effect occurs, and all details will be noted. The outcome measurement will be assessed at 1 and 2 weeks after randomization.

### Sample size estimation

This study aims to evaluate clinical trial feasibility and to investigate basic information about the effects and safety of acupuncture for the treatment of CU, rather than hypothesis testing. Therefore, the sample size was decided based on a rationale for feasibility, precision around the mean and variance, and regulatory considerations and ethical issues that prohibit over-recruitment of participants [41]. Considering an estimated 20% dropout rate, we ensured that the sample size exceeded the minimal number needed to assure the validity of the mean, effect size, and rationale of feasibility. Accordingly, a required sample size of 60 participants was estimated.

### Data collecting and monitoring

The Evidence-based Medicine Center of Chengdu University of Traditional Chinese Data is the Monitoring Committee for Medical Data in this study. Data will be recorded by designated outcome assessors on the paper CRFs, and double-entered into the electronic CRFs, which are established and monitored by the Evidence-based Medicine Center of Chengdu University of Traditional Chinese Medicine. Monitors will audit the data every 3 months. Acupuncturists and statisticians will have no access to these data during the evaluation process.

### Statistical analysis

All data will be presented as means and standard deviations or number (percentage), and all analyses will be based on the intention-to-treat principle. For the description of baseline characteristics, the mean with standard deviation or range with the minimum and maximum values for continuous data and frequency with percentage for dichotomous data will be reported. Homogeneity between the two groups in terms of baseline characteristics will be tested using the two-sample *t* test for continuous data and the chi square (χ2) test for dichotomous data. Analysis of covariance (ANCOVA) or logistic regression will be used for analysis and adjustment of baseline characteristic that differ significantly between the two groups.

The primary outcome of this study is the cumulative UAS in each group. After a normality test, the total score will be compared using the independent *t* test (parametric) or the Mann-Whitney U test (non-parametric) according to the normality of the data. In secondary outcome analysis, the VAS score for itching intensity and the DLQI will be used to analyze the difference, and differences between groups in mean change between baseline and measurements made at 1, 2, 3, and 4 weeks will be analyzed by repeated measures two-factor analysis. The HAMA, HAMD, humoral immunity index, and serum total IgE measurement will be compared using the independent *t* test (parametric) or the Mann-Whitney U test (non-parametric) according to the normality of the data. The χ2 test or logistic regression will be used to analyze dichotomous outcomes. Adverse events will be compared between groups using the χ2 test or Fisher’s exact test. Missing values will be replaced using the method of last observation carried forward. If the participant dropout rate is > 20%, sensitivity analysis will be conducted with the data included.

All statistical tests will be two-sided, and *p* < 0.05 will be considered statistically significant. The statistical software of SPSS (SPSS, SPSS Inc., Chicago, USA) version 21.0 will be used for the analysis.

### Quality control

The trial protocol has been reviewed and revised several times by experts on acupuncture, dermatology, and methodology. Before the trial, all staff are required to attend a series of training sessions. These sessions will ensure that the personnel involved fully understand the research protocol and standard operating procedures for the study. The supervisors will check on case reports and acupuncture procedures twice per month during the trial. The research team will meet regularly (once every 3 months) throughout the trial to discuss progress including recruitment, withdrawals, treatment compliance, and adverse events.

## Discussion

CU is a refractory skin disease with long duration and a high recurrence rate, and acupuncture has been widely used in clinical practice for the treatment of CU in China. However, so far, there has been no appropriately designed randomized controlled trial to provide clear evidence about the effectiveness of acupuncture for the treatment of CU at home and abroad.

Pilot studies, also commonly known as “feasibility” or “vanguard” studies, are comparative randomized trials designed to provide preliminary evidence on the clinical efficacy of a drug or intervention [42, 43]. They are routinely performed in many clinical areas [44–46]. Pilot studies are designed to assess the safety of treatment or interventions, assess recruitment potential, selection of the most appropriate primary outcome measure, determine initial data for the primary outcome measure in order to calculate the sample size for a larger trial, assess the feasibility of international collaboration or coordination in multicenter trials, and increase clinical experience with the study medication or intervention [47, 48]. They are the best way to assess the feasibility of a large, expensive full-scale study. This pilot study aims to evaluate the feasibility of acupuncture treatment for CU and to identify modifications needed in the design of a larger, ensuing hypothesis-testing study.

In this study the inclusion criterion of age range 18–70 years was chosen to cover as wide a range as possible. At the same time, the exclusion of participants taking medication related to CU is essential to obtain accurate clinical results and is determined by considering the possibility of recruitment and ethical factors. Because many patients with CU prefer to go to the dermatology department of the hospital rather than the acupuncture department, we cooperated with the dermatology department of the Affiliated Hospital of Chengdu University of Traditional Chinese Medicine to ensure the required number of recruits. During the study period, when wheals and (or) angioedema are unbearable, rescue medication will be permitted, as we believe that this strategy reflects real-world practice and satisfies ethical obligations.

A standardized treatment protocol will be utilized to assure the reproducibility of the study. In this trial, the treatment protocol was based on traditional acupuncture theory, previous studies [49, 50], guidelines [1] and the consensus of dermatologists and acupuncturists from the West China Hospital of Sichuan University and Affiliated Hospital of Chengdu University of Traditional Chinese Medicine.

The inclusion of a control group allows for a more realistic examination of recruitment, randomization, implementation of interventions, blinded assessment procedures, and retention in blinded interventions [42]. At present, the placebo control of acupuncture includes non-acupoint acupuncture [50], superficial acupuncture [51], and sham acupuncture [52] (the needles do not penetrate the skin). For a long time, there has been controversy about the specific effect of acupuncture: acupuncture works mainly via a placebo effect [53], or tested sham acupuncture controls may not be completely inert [54, 55]. In this trial, the control group will receive superficial acupuncture at non-acupuncture points. The needle depth does not reach the anatomical layers of the acupoints, and the non-acupuncture points are located far from the true acupuncture points. These designs may help minimize nonspecific effects in the control group. In addition, participants in different groups will be treated in different rooms to minimize other nonspecific benefits such as the Hawthorne effect.

The chosen primary outcome is change in the symptom of wheals and angioedema as obtained from the 7-day UAS. Multiple guidelines [1, 15] and a consensus statement [3, 16] recommend the UAS as the main outcome, and the UAS had been widely used as an outcome measure in CU studies. At the same time, assessment of the severity of CU has mainly depended on patient-reported outcomes. The VAS score for itching intensity can directly assess the variations in the severity of subjective symptoms. The available data indicate that urticaria markedly affects both objective functioning and subjective well-being [56–58]. In this trial, we use the DLQI to evaluate quality of life in patients with CU before and after acupuncture treatment. The DLQI is extensively used in evaluating quality of life in skin disease. It is safe and convenient, and also various different language versions of the DLQI are more readily available than the CU Quality of Life Questionnaire (CU-Q2oL) and the Angioedema Quality of Life Questionnaire (AE-QoL). We have obtained the authorization of the Chinese version of DLQI from Finlay [36]. We will also test for correlation between the symptoms of CU and psychiatric disorders by evaluating the HAMD and HAMA in this study. The Humoral immunity index and measurement of serum total IgE will be experimentally assessed to evaluate possible correlation between immunity and the mechanism of the effects of acupuncture on CU.

This trial has its limitations. First of all, this clinical trial will include a small population of participants and will not primarily aim to perform hypothesis testing. For this reason, the results of this study cannot be generalized as basic data for assessing the effect and safety of acupuncture for treatment of CU. Furthermore, because the control group will receive minimal acupuncture, we cannot ignore the possibility of physiological activation caused by needle penetration. The effect of acupuncture will need to be carefully interpreted because the acupuncture points, needling depth, and *de-qi* will be the only differences assessed between groups.

To summarize, this study is funded by the National Key Research and Development Program of the China-Key Project. The study protocol describes the first randomized, patient-blinded and assessor-blinded, sham-controlled trial. The findings of the study will provide feasibility data and basic information about the effect and safety of acupuncture for a future, full-scale RCT in CU.

### Trial status

This trial is currently ongoing. The study commenced on 16 July 2018 and the anticipated end date of the study is 31 March 2019.

## Additional files

Additional file 1: SPIRIT 2013 Checklist: Recommended items to address in a clinical trial protocol and related documents*. (DOC 128 kb)

Additional file 2: Acupuncture treatment details based on the STRICTA 2010 checklist. (DOC 40 kb)

## Data Availability

The study is ongoing. The results of this clinical trial will be shared through scientific articles and academic conferences.

## Abbreviations

AE-QoL: Angioedema Quality of Life Questionnaire
AMCTR: Acupuncture-Moxibustion Clinical Trial Registry
CIU: Chronic inducible urticaria
CRFs: Case report forms
CsA: Cyclosporine A
CSU: Chronic spontaneous urticaria
CU: Chronic urticaria
CU-Q2oL: Chronic Urticaria Quality of Life Questionnaire
DLQI: Dermatology Life Quality Index
EAACI/GA^2^LEN/EDF/WAO: European Academy of Allergology and Clinical Immunology, the Global Allergy and Asthma European Network, World Allergy Organization
ELISA: Enzyme-linked immunosorbent assay
HAMA: Hamilton Anxiety Scale
HAMD: Hamilton Depression Scale
RCT: Randomized controlled trial
SPIRIT: Standard Protocol Items: Recommendations for Intervention Trials
STRICTA: Standards for Reporting Interventions in Clinical Trials of Acupuncture
UAS: Urticaria Activity Score
VAS: Visual analogue scale
