# Supplementary material for: Efficacy and safety of acupuncture for patients with chronic urticaria: study protocol of a randomized, sham-controlled pilot trial
Source: Trials. 2019 Jun 4;20:326. doi: 10.1186/s13063-019-3433-1 (PMC6549330; doi:10.1186/s13063-019-3433-1)
Supplement: Supplementary file 2 — Acupuncture treatment details based on the STRICTA 2010 checklist. (DOC 40 kb) [file 13063_2019_3433_MOESM2_ESM.doc]

**Additional 2** Acupuncture treatment details based on the STRICTA 2010 checklist

| **Item** | **Item number** | **Detail** |
| --- | --- | --- |
| **1.Acupuncture rationale** | 1a) Style of acupuncture | Traditional Chinese Medicine |
|  | 1b)Reasoning for treatment | The treatment protocol was based on traditional acupuncture theory, previous studies guidelines and the consensus of dermatologists and acupuncturists from West China Hospital of Sichuan University and Affiliated Hospital of Chengdu University of Traditional Chinese Medicine. |
|  | 1c)Extent to which treatment was varied | All participants will receive standardized treatment. |
| **2. Details of needling** | 2a) Number of needle insertions per subject per session | 13 |
|  | 2b) Names of points used | Eight fixed points:GV20、GV24、LI11、CV12、ST25、SP10、ST36、SP6. |
|  | 2c)Depth of insertion, based on a specified unit of measurement | From 0.5 to 1.5 cun |
|  | 2d) Response sought (e.g., de qi or muscle twitch response) | De qi sensation |
|  | 2e) Needle stimulation (e.g., manual, electrical) | Manual stimulation: needle rotation with thumb and index fingers at 3 Hz |
|  | 2f) Needle retention time | 30 min |
|  | 2g) Needle type | Sterile disposable stainless steel needles of various lengths and diameters (Huatuo Medical Instruments Co. Ltd., Suzhou, China; 0.3 mm × 25 mm/0.3 mm × 40 mm) |
| **3. Treatment regimen** | 3a)Number of treatment sessions | Ten treatment sessions in both acupuncture and sham acupuncture groups. |
|  | 3b)Frequency and duration of treatment sessions | 1 sessions per day for 5 days. |
| **4. Other components of treatment** | 4a)Details of other interventions administered to the acupuncture group (e.g., moxibustion, cupping, herbs,exercises, lifestyle advice) | No other interventions during the study period were allowed. Light diet and do not eat beef, lamb, seafood, mushrooms,and so on. |
|  | 4b) Setting and context of treatment, including instructions to practitioners, and information and explanations to patients | The study will be conducted at the famous doctor's medical center of Chengdu University of Traditional Chinese Medicine. All patients will be admitted to the acupuncture therapeutic room for treatment. All information except patient allocated group will be provided to participants. |
| **5.Practitioner background** | 5)Description of participating acupuncturists | Acupuncturists who are registered Chinese Medicine Practitioners in China and have at least 3 years’ clinical experience in acupuncture practice. Besides, they went through training classes before this trial, and simulation to ensure that they are able to provide identical acupuncture treatment in accordance with a pre-defined protocol. |
| **6. Control interventions** | 6a)Rationale for the control or comparator in the context of the research question, with sources that justify this choice | Based on traditional acupuncture theory, previous studies, and the consensus of acupuncturists from Chengdu University of Traditional Chinese Medicine. |
|  | 6b) Precise description of the control or comparator. If sham acupuncture or any other type of acupuncture-like control is used, provide details as for Items 1 to 3 above. | For the control group: participants will receive superficial non-acupoint acupuncture 10 sessions over 2 weeks. The same type of acupuncture needle will be inserted perpendicular to the skin at a depth of 1.0-3.0 mm and the needles will be retained for 30 minutes without any manipulation to avoid the deqi sensation as much as possible. |

**Abbreviation:** GV: Governor; LI: large intestine; ST: stomach; SP: spleen; CV: conception vessel; STRICTA: Standards for Reporting Interventions in Clinical Trials of Acupuncture.
